# The Imbalance in Serum Concentration of Th-1- and Th-2-Derived Chemokines as One of the Factors Involved in Pathogenesis of Atopic Dermatitis

**DOI:** 10.1155/2009/269541

**Published:** 2009-07-22

**Authors:** Joanna Narbutt, Aleksandra Lesiak, Anna Sysa-Jedrzeiowska, Marcin Zakrzewski, Jarosław Bogaczewicz, Iwona Stelmach, Piotr Kuna

**Affiliations:** ^1^Department of Dermatology, Medical University of Lodz, 94-017 Lodz, Poland; ^2^Department of Pediatrics and Allergy, Medical University of Lodz, 90-153 Lodz, Poland; ^3^Division of Internal Medicine, Asthma and Allergy, Medical University of Lodz, 93-036 Lodz, Poland

## Abstract

Atopic dermatitis (AD) is an inflammatory skin disease in which pathogenesis chemokines are partially involved. The aim of the paper was to assess the serum level of CXCL-9, CXCL-10, CXCL-11, CXCL-12, CCL-17, CCL-20, CCL-21, CCL-22, CCL-27, and IL-18 chosen in AD patients by ELISA assay. Forty patients (mean age 11.4 years old) with AD and 50 healthy controls were enrolled into the study. The patients and controls were divided into two age categories: under 10 years old (Group 1 and Control 1) and over 10 years old (Group 2 and Control 2). Significantly lower serum concentration of CXCL-9, CXCL-10, CCL-17, and IL-18 and higher concentration of CXCL-12 and CCL-27 were found in Group 1 when compared to Control 1. In Group 2 serum concentration of CXCL-12, CCL-17, CCL-22 was higher than in Control 2. The obtained results indicate the imbalance in chemokine serum levels in AD what suggests their role in the disease pathogenesis.

## 1. Introduction

Atopic dermatitis (AD) is a chronic and recurrent disease which concerns 10–20% of population [1]. The onset of AD is usually before 2 years old, and in approximately 60% of the patients skin lesions of different intensity remain for the whole life [2]. AD is characterized by typical morphology and distribution of skin lesions, severe pruritus, and familial atopic history. Clinical phenotype of the disease depends on multiple interactions between genetic and immunological disturbances, epidermal barrier impairment, and environmental factors [3, 4]. 

Histopathologically skin lesions present mainly with a dermal infiltrate of mainly CLA+ memory T cells and Langerhans cells [5]. Leukocyte trafficking into the skin in AD patients is probably mainly regulated by adhesion molecules and chemoattractive proteins, so-called chemokines [6, 7]. Chemokines are small secreted proteins involved in migration and activation of lymphocytes T. Recently published papers indicate their role in pathogenesis of multiple inflammatory skin diseases including atopic dermatitis [8]. 

In active skin lesions in the course of AD infiltrates of Th-2 lymphocytes releasing IL-4 and/or IL-13 are found. These cells express CCR4, CCR8, and CRTH2 receptors on their surface. Lymphocytes Th-2 migration is selectively induced by such chemokines as CCL17 and CCL22, which are highly overexpressed on the keratinocytes in AD patients epidermis. These phenomena lead in a consequence to development of local inflammation [9, 10]. In the studies analyzing serum concentration of these chemokines, increased levels of CCL-17 and CCL-22 were found in AD patients, and their concentrations strongly correlated with disease activity [11, 12]. 

Shimada et al. [13] found increased levels of Th-2 (CCL-17 and CCL-22) and Th-1 (CXCL-9) chemokines in AD patients. They also found a positive correlation between Th-2 chemokines serum level and total IgE concentration, moreover Th-2 chemokines correlated with Th-1 ones. In another study performed in infantile AD patients (mean age 4.5 months) also increased levels of CCL-17, CCL-20, CCL-27 were observed which strongly correlated with disease activity, however the most prominent correlation was observed for CCL-27 [14]. 

Interleukin (IL)-18 is involved in pathogenesis of type-2 helper cells-mediated diseases including atopic dermatitis. According to literature, its serum concentration is significantly elevated in AD patients and correlates with clinical severity of the disease [15, 16].

Most literature data point out an important role of chemokine network imbalance in development of atopic dermatitis, however there are scarce data on the complex analysis of serum levels of Th-1- and Th-2-derived chemokines in AD patients. Thus, the aim of the paper was to assess the serum level of CXCL-9, CXCL-10, CXCL-11, CXCL-12, CCL-17, CCL-20, CCL-21, CCL-22, CCL-27, IL-18 in two AD patient groups, below and over 10 years old. Additionally we analyzed serum levels of the chosen chemokines in two groups of healthy volunteers, aged-matched, to note any age-dependent variations in healthy population.

## 2. Material and Methods

### 2.1. Patients

Forty patients (mean age 11.4 years old; 23 F, 15 M) with AD and 50 healthy controls, age and sex matched were enrolled into the study. AD was diagnosed according to criteria proposed by Hanifin and Rajka [17]. The enrolled patients were divided into two age categories: under 10 years old (Group 1; *n* = 23) and over 10 years old (Group 2; *n* = 17). According to this criterion, the control group was divided as well (Control 1 under 10 years old *n* = 30; and Control 2 over 10 years old *n* = 20). We used criterion of 10 years old as a cut-off point because this age is believed to initiate adolescence life period. Clinical characteristics of the patients are presented in Table 1.

### 2.2. Methods

Each patient or his/her parents gave written informed consent before entering the study, and all the experiments were approved by the Local Ethics Committee. The investigations were carried out in accordance with Declaration of Helsinki. Before entering the study the subjects underwent thorough physical examination, and scoring atopic dermatitis (SCORAD) index was assessed [18]. The patients enrolled to the study had moderate AD (mean SCORAD index 23 range 16–39). Serum samples were analyzed for CXCL-9, CXCL-10, CXCL-11, CXCL-12, CCL-17, CCL-20, CCL-21, CCL-22, CCL-27, IL-18 concentration with ELISA assay (R&D system, Mineapolis, Minn, USA) according to manufacturer's instructions. The concentrations were calculated from the standard curve generated by curve-fitting programme.

## 3. Statistical Analysis

Data were analyzed using the Mann-Whitney U test, and correlations coefficients were determined by using the Spearman rank correlation test. A *P*-value of <.05 was considered as statistically significant.

## 4. Results

Median concentrations of all the analyzed proteins: CXCL-9, CXCL-10, CXCL-11, CXCL-12, CCL-17, CCL-20, CCL-21, CCL-22, CCL-27, IL-18 are presented in Table 2.

### 4.1. The Median Concentration of CXCL-9, CXCL-10, CXCL-11, CXCL-12, CCL-17, CCL-20, CCL-21, CCL-22, CCL-27, IL-18 in AD Patients below 10 Years Old (Group 1)

The median serum CXCL-9, CXCL-10, CCL-17, and IL-18 level was statistically significantly lower in AD patients from Group 1 when compared to the Control 1 (56.7 pg/mL versus 87.3 pg/mL; *P* = .003; 84.8 pg/mL versus 98.0 pg/mL; *P* = .04; 405.2 pg/mL versus 620.1 pg/mL, *P* = .04; 64.8 pg/mL versus 94.7 pg/mL, *P* = .0001; resp.). In Group 1 the median serum concentration of CXCL-12 and CCL-27 was significantly higher than that in the Control 1 group (2444.9 pg/mL versus 2135.8 pg/mL, *P* = .004; 463.5 pg/mL versus 406.6 pg/mL, *P* = .03; resp.). For other chemokines median serum values did not differ statistically when compared to the Control 1 (*P* > .05 for all comparisons).

### 4.2. The Median Concentration of CXCL-9, CXCL-10, CXCL-11, CXCL-12, CCL-17, CCL-20, CCL-21, CCL-22, CCL-27, IL-18 in AD Patients over 10 Years Old (Group 2)

The median serum CXCL-12, CCL-17, and CCL-22 levels were significantly higher in AD patients from Group 2 than in the age-matched Control 2 (2553.5 pg/mL versus 2361.1 pg/mL, *P* = .01; 357.6 pg/mL versus 178.3 pg/mL, *P* = .04; 1152.5 pg/mL versus 606.1 pg/mL, *P* = .001; resp.). For other chemokines median serum values did not differ statistically when compared to the Control 2 (*P* > .05 for all comparisons).

### 4.3. Differences in the Median Concentration of CXCL-9, CXCL-10, CXCL-11, CXCL-12, CCL-17, CCL-20, CCL-21, CCL-22, CCL-27, IL-18 between Groups 1 and 2

Comparing serum median levels of analyzed chemokines between two AD groups (Group 1 versus Group 2) the only significant difference was found for CCL-20 which median value was higher in children below 10 years old than in older population (Group 2) (7.8 pg/mL versus 7.4 pg/mL; *P* = .03).

### 4.4. Differences in the Median Concentration of CXCL-9, CXCL-10, CXCL-11, CXCL-12, CCL-17, CCL-20, CCL-21, CCL-22, CCL-27, IL-18 between Controls 1 and 2

Comparing CXCL-9, CXCL-11, CCL-17, CCL-20, CCL-22, and IL-18 serum median concentration between Controls 1 and 2 we found significantly higher values in younger population (Control 1) than in Control 2 (87.3 pg/mL versus 17.9 pg/mL, *P* = .00004; 66.9 pg/mL versus 43.3 pg/mL, *P* = .049; 620.1 pg/mL versus 178.3 pg/mL, *P* = .001; 8.3 pg/mL versus 6.9 pg/mL, *P* = .008; 1410.2 pg/mL versus 606.1 pg/mL, *P* = .00003; 94.7 pg/mL versus 97.2 pg/mL, *P* = .049; resp.). For other chemokines median serum values did not differ statistically when compared two control groups (*P* > .05 for all comparisons).

### 4.5. Correlations between Median Serum Levels of CXCL-9, CXCL-10, CXCL-11, CXCL-12, CCL-17, CCL-20, CCL-21, CCL-22, CCL-27, IL-18 in Analyzed Groups

Positive correlation between median serum concentration of CXCL-11 and CXCL-9 (*r* = 0.44, *P* = .03), CXCL-9 and CXCL-10 (*r* = 0.42, *P* = .04), and CXCL-10 and IL-18 (*r* = 0.8, *P* = .000001) was found in AD patients below 10 years old (Group 1).

In Group 2 (AD patients over 10 years old) we found positive correlations of the median serum levels of the analyzed proteins for the following parameters: CXCL-11 and CXCL-9 (*r* = 0.85, *P* = .00001), CXCL-11 and CXCL-10 (*r* = 0.8, *P* = .00006), CXCL-11 and CCL-20 (*r* = 0.96, *P* < .001), CXCL-9 and CXCL-10 (*r* = 0.59, *P* = .01), CXCL-9 and CCL-20 (*r* = 0.84, *P* = .00002), CXCL-10 and CCL-20 (*r* = 0.79, *P* = .0002), and CCL-17 and IL-18 (*r* = 0.5, *P* = .03). Moreover in this group we observed a positive correlation between serum median concentration of CCL-22 and patients' age (*r* = 0.52, *P* = .003).

In Control 1 we found positive correlations between serum levels of the following chemokines: CXCL-11 and CCL-21 (*r* = 0.4, *P* = .03), CXCL-11 and CCL-22 (*r* = 0.37, *P* = .04), CXCL-11 and CCL-17 (*r* = 0.36, *P* = .048), CXCL-9 and CXCL-10 (*r* = 0.47, *P* = .008), CCL-17 and CCL-22 (*r* = 0.68, *P* = .00003). Contrary, negative correlation between serum level of CCL-21 and CXCL-9 (*r* = −0.36, *P* = .04) was noted. Analysing correlation between serum chemokine levels and patients' age we found a negative link for CCL-21 and CCCL-22 (*r* = −0.5, *P* = .004; *r* = −0.36, *P* = .048; resp.).

In the Control 2 positive correlations were found only between CXCL-10 and CCL-21, and CXCL-17 and CCL-20 (*r* = 0.84, *P* = .002; *r* = 0.9, *P* = .0003; resp.).

Taking the whole AD group (Group 1 and 2) into statistical analysis we found no correlation between serum levels of analyzed parameters and patients' age, while doing the same analysis for whole control group (Control 1 + Control 2) we found negative correlations between age of the subjects and the following proteins: CCL-17, CCL-22 and IL-18 (*r* = −0.38, *P* = .01; *r* = −0.67, *P* = .000002; *r* = −0.3, *P* = .045; resp.).

### 4.6. Correlations between Median Serum Levels of CXCL-9, CXCL-10, CXCL-11, CXCL-12, CCL-17, CCL-20, CCL-21, CCL-22, CCL-27, IL-18 and IgE Concentration and Eosinophilia in Analyzed Groups

In Group 1 a positive correlation between mean total IgE serum concentration and CCL-20 was found (*r* = 0.52, *P* = .009), while in Group 2 it was found for CCL-17 (*r* = 0.68, *P* = .002), CCL-27 (*r* = 0.049, *P* = .046) and IL-18 (*r* = 0.58, *P* = .01). Analysing all the subjects from AD group (Group 1 and 2) IgE serum concentration correlated positively only with CCL-17 (*r* = 0.49, *P* = .01).

In AD patients below 10 years old (Group 1) a positive correlation between eosinophilia and CCL-20 (*r* = 0.53, *P* = .009) and IL-18 (*r* = 0.45, *P* = .03) was noted while in Group 2 eosinophilia correlated positively with CCL-27 (*r* = 0.52, *P* = .03). Analyzing all the subjects from AD group (Groups 1 and 2) eosinophilia correlated positively only with CCL-22 (*r* = 0.36, *P* = .02).

## 5. Discussion

Chemokines are small proteins which contribute to leukocytes trafficking into the skin. CXCL-9, CXCL-10, and CXCL-11 recruit lymphocytes mainly to Th-1 type inflammatory sites while chemokines such as CCL-11, CCL-17, CCL-22 lead to Th-2 dominated pattern of cell recruitment [19]. CCL-27 selectively attracts CLA + memory T cells via CCR-10 receptor expressed on these cells [20, 21].

Recent data indicate increased serum level of CCL-27 in AD patients, correlating with disease activity, what suggests its role in inflammatory process [22, 23]. Hon et al. [23] assessed CCL-27 serum level in children aged 1–11 years with mean SCORAD [29.7] and found its higher serum concentration, what is in line with our results, however contrary to the authors we found no correlation between CCL-27 and CCL-17 and CCL-22 concentrations. Also level of CCL-17 in Group 1, although statistically different than that in Control 1, was lower in AD patients than in healthy ones. Such discrepancy between our results and published ones in other papers, indicates that it is still unclear either increased or decreased CCL-17 level is a characteristic for AD patients, however its distinct level when compared to the control groups testifies its role in AD pathogenesis. In our study similar observations concern CXCL-9, CXCL-10, and IL-18. 

Interestingly, in Group 2 (patients over 10 years old) we found elevated serum levels of CCL-17, CCL-22, and CXCL-12 what also proves their role in AD pathogenesis. Other authors also showed significantly higher concentrations of CCL-17, CCL-22, and eotaxin in AD patients than in healthy control. They found positive correlations between serum level of CCL-17 and CCL-22 and total IgE concentration and as well these chemokines correlated positively with SCORAD index [24]. In our study CCL-17, CCL-27 and IL-18 serum levels correlated positively with total IgE in Group 2, and eosinophilia correlated positively with CCL-27. Hon et al. [25] revealed correlation between IgE and CCL-17 and eosinophiles count but not with CCL-27 what is only partially consistent with our results.

In skin biopsies taken from AD patients CCL-20 expression is observed in basal layer of epidermis. This protein is a strong chemoattractant for immature dendritic cells and memory T cells via interactions with CCR6. CCL20 may be induced on keratinocytes under proinflammatory cytokines such: IL-1*α* or TNF-*α*. In healthy epidermis CCL20 is constitutively expressed in epidermal basal layer, however its expression is significantly lower than in inflammatory skin [26, 27]. These data prove CCL-20 role in AD pathogenesis. Although we found no differences between CCL-20 serum concentration in AD patients and controls, its higher concentration was observed in Group 1 than in Group 2. Moreover, in younger population (Group 1) IgE and eosinophilia correlated with CCL-20. To our knowledge, there are no data in literature on the subjects, however observed association and data mentioned above provide its role in AD pathogenesis in younger population and its level normalization in line with the age. 

To our knowledge, there are no data on CCL-21 serum levels in AD patients. We examined this chemokine as it is strongly chemoattractive to lymphocytes T, enhances expression of LFA-1 on these cells, and mediates cell-to-cell adhesion. Besides, CCL-21 and CCR7 receptor influence naive T cell migration to lymph nodes where antigen is presented. In healthy skin immunostaining for CCL21 is negative, however it is expressed on dermal endothelial cells in atopic dermatitis [28]. Weninger et al. [29] showed that CCL21 expression on blood vessels positively correlated with the presence of CD45RA+ T cells in the inflammatory infiltrate. Although CCL21 expression was found in inflammatory T cell-mediated diseases, its exact role in their pathogenesis is not elucidated. Our study in which we found no differences in CCL-21 serum concentration between AD patients and controls does not prove the role of the chemokine in AD pathogenesis. 

To assess chemokines serum levels depending on age in healthy population, we attempted to check differences between Controls 1 and 2. Our analysis revealed a distinct pattern in healthy population than that in AD groups. In younger healthy population we found increased levels of CXCL-9, CXCL-11, CCL-17, CCL-20, CCL-22, and IL-18 while comparing Groups 1 and 2 the only significance concerned CCL-20. 

 In the study published by Furusyo et al. [30] no differences in serum level of CCL-17 between AD patients (children 0–5 years old) and age-matched healthy control were revealed. Moreover in healthy children they observed that serum CCL-17 concentration decreased with age while serum CCL-17 in AD patients did not differ in relation to age. The authors revealed strong dependence between CCL-17 serum concentration and AD course during childhood. These data are partially consistent with ours, as we also observed a decrease in CCL-17 with age in healthy population and no such age-dependence in AD group. To our knowledge there are no more data analyzing chemokines in this aspect. Lack of these naturally occurring changes in chemokine serum levels in AD patients provides their role in the disease pathogenesis. This hypothesis may be partially proven by our observation on the lack of correlation between age and chosen chemokines serum levels in AD patients and the presence of multiple negative correlations between CCL-17, CCL-22, IL-18, and age in the whole control group. 

 Concluding, we may assume that in younger children with AD a decreased serum level of Th-1-derived chemokines is one of the factors involved in the disease development. The imbalance between Th-1 and Th-2 is probably involved in AD pathogenesis as well, what in our paper is especially emphasized by differences in chemokine concentration between two AD groups and two age-matched controls. Our study, similar to others, revealed significant changes between chemokine levels in AD patients and controls, however not always consistent with other authors what may result from two main reasons. The first one is a new aspect of AD pathogenesis, mostly focused now on the impairment of epidermal barrier and innate immune defense as the primary causative factors involved in AD; only these disturbances lead secondary to induction of adaptive immune response, inflammation development involving chemokines disturbances. The second reason may be the lack of objective and standardized method for AD clinical evaluation, thus the patients enrolled to the studies in different centers, although with the same SCORAD index, may have a little different clinical picture. 

 Based on literature and our results we conclude that chemokine imbalance is involved in AD pathogenesis, however discrepancies obtained in many studies and relatively small number of the patients included in our study do not allow to draw equivocal conclusions. In our opinion further studies correlating chemokine serum levels, their expression in the skin, and AD clinical picture are required and probably will give new light on the disease pathogenesis.

## Figures and Tables

**Table 1:** Clinical chatacteristics of AD patients.

Clinical feature	Group 1; *n* = 23	Group 2; *n* = 17
Sex	11 F, 12 M	11F, 6 M
Age (yr)	7.1	17.1
Disease onset	Before 6 month *n* = 10	Before 6 month *n* = 5
	6 month-2 yr *n* = 11	6 month-2 yr *n* = 10
	Over 3 yr *n* = 2	Over 3 yr *n* = 2
Dry skin	Yes *n* = 20	Yes *n* = 17
	No *n* = 3	No *n* = 0
Keratosis pilaris	Yes *n* = 15	Yes *n* = 8
	No *n* = 8	No *n* = 9
Hand and foot dermatitis	Yes *n* = 4	Yes *n* = 2
	No *n* = 19	No *n* = 15
Ichtyosis	Yes *n* = 0	Yes *n* = 0
	No *n* = 23	No *n* = 17
Nipple eczema	Yes *n* = 0	Yes *n* = 0
	No *n* = 23	No *n* = 17
Cheilitis	Yes *n* = 12	Yes *n* = 11
	No *n* = 11	No *n* = 6
Recurrent conjunctivitis	Yes *n* = 12	Yes *n* = 11
	No *n* = 11	No *n* = 6
Denni-Morgan infraorbital fold	Yes *n* = 19	Yes *n* = 13
	No *n* = 4	No *n* = 4
Orbital darkening	Yes *n* = 16	Yes *n* = 16
	No *n* = 7	No *n* = 1
Pityriasis alba	Yes *n* = 4	Yes *n* = 6
	No *n* = 19	No *n* = 11
Anterior Neck folds	Yes *n* = 3	Yes *n* = 3
	No *n* = 20	No *n* = 14
White dermographism	Yes *n* = 9	Yes *n* = 2
	No *n* = 14	No *n* = 15
Palmar hyperlinearity	Yes *n* = 3	Yes *n* = 9
	No *n* = 20	No *n* = 8
Itch when sweating	Yes *n* = 17	Yes *n* = 10
	No *n* = 6	No *n* = 7
Intolerance to wool	Yes *n* = 22	Yes *n* = 15
	No *n* = 1	No *n* = 2
Course influence by environment al and emotional factors	Yes *n* = 16	Yes *n* = 9
	No *n* = 7	No *n* = 8
Family history of atopy	Yes *n* = 15	Yes *n* = 11
	No *n* = 8	No *n* = 6
Clinical remission	Yes *n* = 11	Yes *n* = 13
	No *n* = 12	No *n* = 14
Mean IgE concentration (IU/mL)	173.46	257.27
% eosinophilia	4.3	3.7

**Table 2:** Median serum concentration of analyzed chemokines in AD patients (Group 1 and Group 2) and Controls (Control 1 and 2).

Protein	Median concentration pg/mL (upper/lower quartile)	Median concentration pg/mL (upper/lower quartile)	Mdian concentration pg/mL (upper/lower quartile)	Median concentration pg/mL (upper/lower quartile)	*P* (statistically significant)
	Group 1 (1) *n* = 23	Control 1 (2) *n* = 30	Group 2 (3) *n* = 17	Control 2 (4) *n* = 20	
CXCL-9	56.7 (29.8/75.1)	87.3 (55.4/132.0)	34.1 (23.9/62.9)	17.9 (12.5/26.7)	*P*_1−2_ = .003
					*P*_2−4_ = .00004
CXCL-10	84.8 (60.8/94.3)	98.0 (76.4/135.2)	93.6 (67.4/114.9)	75.8 (63.2/112.5)	*P*_1−2_ = .04
CXCL-11	52.9 (36.1/64.6)	66.9 (40.8/135.2)	57.4 (45.5/97.6)	43.2 (36.1/57.4)	*P*_2−4_ = .049
CXCL-12	2444.9 (2291.2/2711)	2135.8 (1821.4/2364.7)	2553.5 (2393.9/2618.2)	2361.1 (2239.5/2430.3)	*P*_1−2_ = .004
					*P*_3−4_ = .01
CCL-17	405.2 (294.4/729.9)	620.1 (369.9/889.2)	357.6 (257.4/547.3)	178.3 (132.8/304.9)	*P*_1−2_ = .04
					*P*_2−4_ = .001
					*P*_3−4_ = .04
CCL-20	7.8 (7.6/13.1)	8.3 (7.8/35.1)	7.4 (7.1/7.9)	6.9 (5.9/7.8)	*P*_1−3_ = .03
					*P*_2−4_ = .008
CCL-21	158.1 (108.8/234.9)	175.5 (85.5/243.3)	158.1 (126.9/209.8)	160.7 (140.4/188.4)	*P* < .05 for all comparisons
CCL-22	1147.5 (908.6/1395.3)	1410.2 (1010.5/1612.8)	1152.5 (945.3/1390.5)	606.1 (571.3/726.1)	*P*_2−4_ = .00003
					*P*_3−4_ = .001
CCL-27	463.5 (406.1/519.6)	406.6 (326.8/452.9)	487.1 (367.1/628.3)	385.0 (342.7/544.3)	*P*_1−2_ = .03
IL-18	64.8 (54.1/73.1)	94.7 (80.6/124.9)	63.3 (56.9/83.7)	77.2 (60.4/96.3)	*P*_1−2_ = .0001
					*P*_2−4_ = .049
